# Synthesis and crystal structure of 4,6-di­amino-3-(prop-2-en-1-yl)-2-sulfanyl­idene-2,3-di­hydro-1,3,5-triazin-1-ium chloride

**DOI:** 10.1107/S2056989026008182

**Published:** 2026-08-14

**Authors:** Reham A. Mohamed-Ezzat, Benson M. Kariuki, Galal H. Elgemeie

**Affiliations:** a Chemistry of Natural & Microbial Products Department, Pharmaceutical and Drug Industries Research Institute, National Research Centre, Cairo, Egypt; b School of Chemistry, Cardiff University, Main Building, Park Place, Cardiff CF10 3AT, United Kingdom; c Department of Chemistry Faculty of Science Capital University, Cairo,Egypt; Tokyo University of Science, Japan

**Keywords:** crystal structure, synthesis, triazine­thione, Hirshfeld surface

## Abstract

The title structure, C_6_H_10_N_5_S^+^·Cl^−^, comprises two independent 4,6-di­amino-3-(prop-2-en-1-yl)-2-sulfanyl­idene-2,3-di­hydro-1,3,5-triazin-1-ium cations and two chloride anions. The propene groups assume different conformations in the two cations. The two independent cations are linked by two N—H⋯N hydrogen bonds, forming *R*^2^_2_(8) motifs. These hydrogen-bonded cation pairs are in turn bridged by the chloride anions through N—H⋯Cl contacts to neighbouring pairs.

## Abstract 2

In the title compound, the cations are hydrogen bonded in pairs, which are bridged by the chloride anions through N—H⋯Cl hydrogen bonding.

## 1. Chemical context

Triazine is a privileged heterocyclic scaffold for the synthesis of a broad spectrum of pharmacologically bioactive compounds. 1,3,5-Triazine analogs have displayed a wide range of biological potencies, including anti­fungal, anti­bacterial, anti-inflammatory, anti­cancer, anti-Alzheimer’s and anti­viral properties (Patil *et al.*, 2020 ▸; Kumawat *et al.*, 2024 ▸).

Structural modification of the triazine nucleus through substitution at different ring positions provides access to a diverse range of functionalized derivatives suitable for further chemical transformation. The incorporation of an allyl substituent in the triazine core further expands the synthetic utility of triazine-2-thione derivatives.

Building upon the therapeutic significance of the triazine core, the incorporation of allyl-containing functionalities has attracted considerable attention. The allyl moiety is a key structural motif widely incorporated in natural products and bioactive mol­ecules. Integration of an allyl group can modulate mol­ecular flexibility, lipophilicity, and binding inter­actions, thereby modifying the pharmacological profile of the compound. Besides acting as a protecting or functional group, the allyl scaffold presents potential for further chemical modification via oxidation, cyclization, and various reactions. Numerous allyl-containing analogs have demonstrated a range of biological potencies. Consequently, the allyl fragment has been extensively utilized in medicinal chemistry as a valuable and versatile pharmacophore for the development of novel therapeutic agents (Astrain-Redin *et al.*, 2023 ▸).

In addition to allyl functionality, sulfur is a prevalent heteroatom in anti­metabolic heterocycles (*e.g.* Mohamed-Ezzat *et al.*, 2026 ▸, Mohamed-Ezzat & Elgemeie, 2023 ▸; Elgemeie *et al.* 1999 ▸, 2015 ▸; Elgemeie & Mohamed, 2014*a* ▸,*b* ▸; Mustafa *et al.*, 2022 ▸). Thione functionality serves as a synthetic precursor for the construction of various sulfur containing heterocycles via nucleophilic transformation reactions and tautomeric thione-thiol inter­conversion. These unique characteristics have established thione-incorporated heterocycles as remarkable structural motifs in drug discovery and development (Katritzky *et al.*, 2008 ▸; Mustafa *et al.*, 2022 ▸).

Following our recently-described approach to the synthesis of triazines (Mohamed-Ezzat & Elgemeie, 2024*a* ▸,*b* ▸; Mohamed-Ezzat *et al.*, 2024 ▸, 2025 ▸), particularly triazine­thione (Ahmed *et al.* 2026 ▸), and in a continuation of our research to explore the synthetic potential of this scaffold and to expand the chemical diversity of triazine systems by incorporating the allyl and thione functionalities into the triazine scaffold, we now report the synthesis and structure of the title compound (**I**).
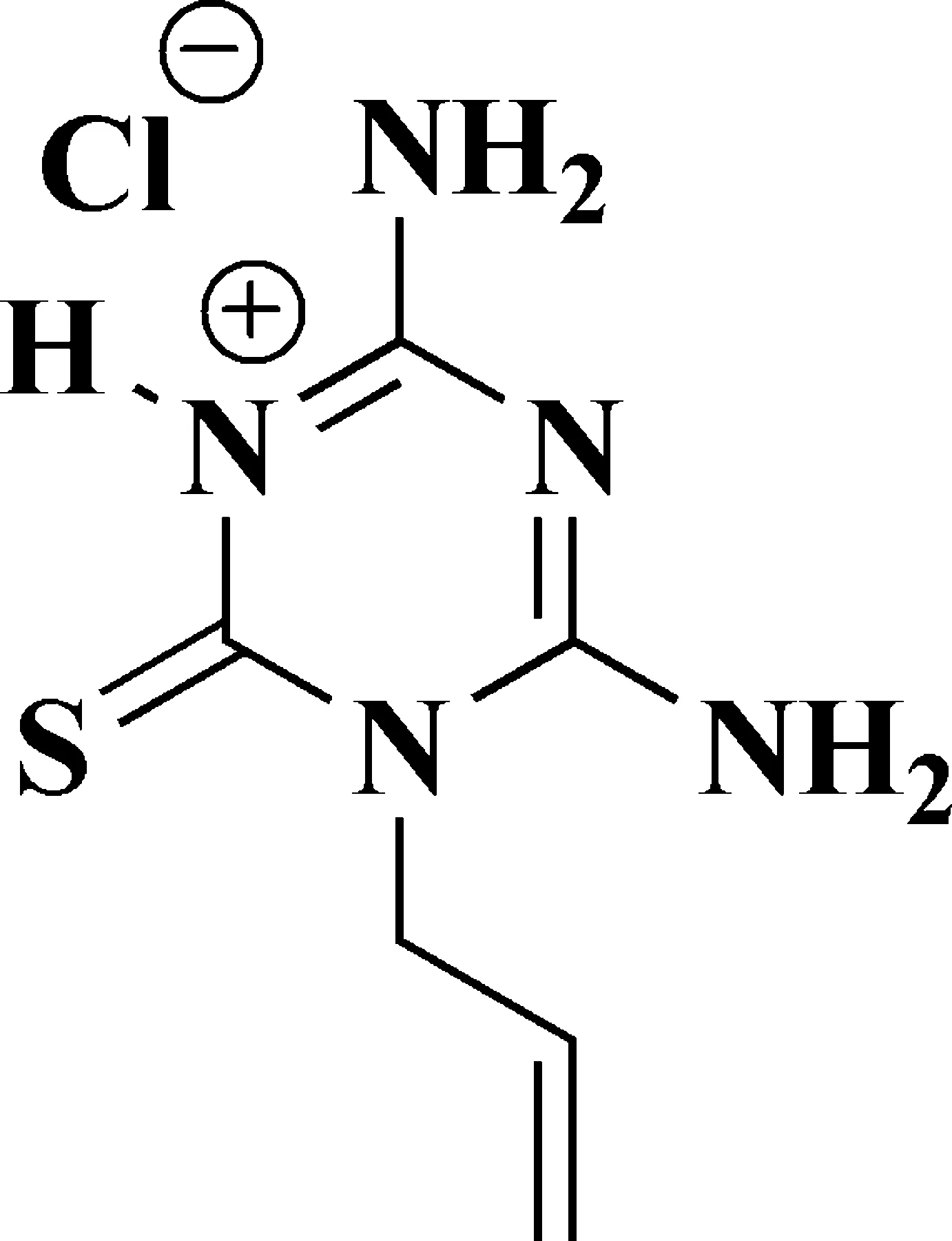


## 2. Structural commentary

**Figure 1:**
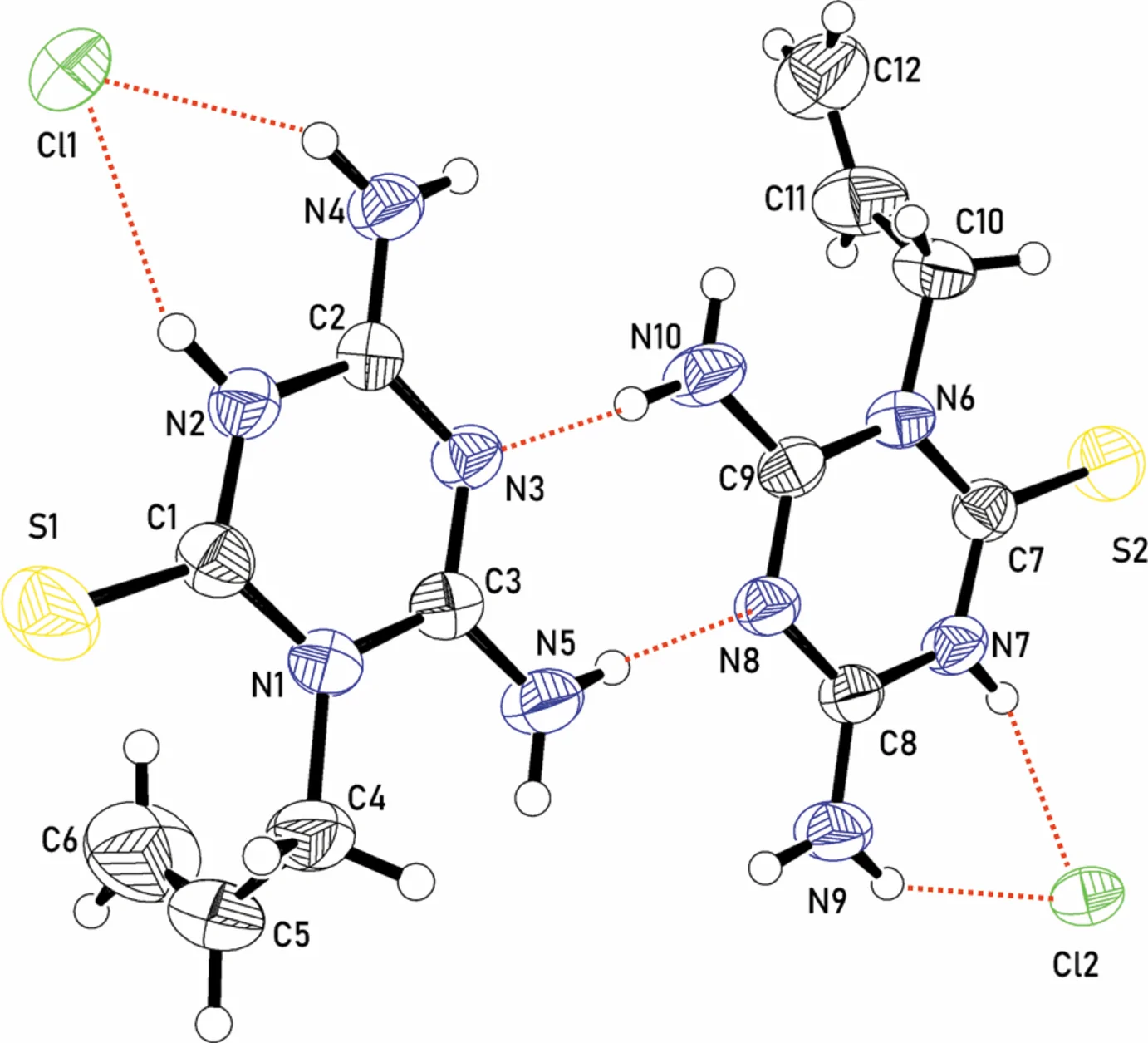
The asymmetric unit of the title compound showing displacement ellipsoids at the 50% probability level. Hydrogen bonds are indicated by dashed lines

The asymmetric unit of (**I**) contains two independent 4,6-di­amino-3-(prop-2-en-1-yl)-2-sulfanyl­idene-2,3-di­hydro-1,3,5-triazin-1-ium cations and two chloride anions (Fig. 1 ▸). Each cation consists of a di­amino­triazine­thione (DTT) group (C1–C3/N1–N5/S1) and C7–C9/N6–N10/S2 for the two independent cations, here referred to as **c1** and **c2**, linked to a propene group (C4–C6 and C10–C12 for **c1** and **c2**, respectively). The DTT groups are almost planar in the two cations but the orientations of the propene groups differ with torsion angles N1—C4—C5—C6 and N6—C10—C11—C12 of 6.9 (6)° and 147.5 (3)° for **c1** and **c2**, respectively. The corresponding bond lengths and angles are similar in two cations apart from the propene groups where C4—C5 [1.470 (5) Å] is slightly shorter than C10—C11 [1.486 (4) Å] and the angle C4—C5—C6 [127.3 (3)°] is greater than C10—C11—C12 [124.2 (4)°]. One nitro­gen atom of each independent triazine ring (N2 in **c1** and N7 in **c2**) is protonated. The C—N bond lengths associated with the protonated nitro­gen atoms (C1—N2, C2—N2, C8—N7 and C7—N7) are in the range 1.355 (3) to 1.362 (3) Å. In contrast, the bond lengths (C2—N3, C3—N3, C8—N8, C9—N8) for the unprotonated nitro­gen atoms (N3 for **c1** and N8 for **c2**) are shorter, being in the range 1.328 (3)–1.331 (3) Å. The associated bond angles for the protonated nitro­gen atoms [C1—N2–C2 = 123.7 (2) in **c1** and C7—N7—C8 = 123.7 (2)° for **c2**] are greater than those for the unprotonated atoms [C2—N3—C3 = 117.08 (19)° in **c1** and C8—N8—C9 = 117.41 (19)° in **c2**]. The di­amino­triazine­thione groups in the structure of *cis*(4,6-di­amino-2-thiono-1*H*-(1,3,5)triazinium)aquabis­(oxalato-*O*,*O*′)dioxouranium(VI) *N*-cyan­o­guanidine (Serezhkina *et al.*, 2007 ▸) show a similar contrast in the geometry around the protonated and unprotonated nitro­gen atoms.

## 3. Supra­molecular features

**Figure 2:**
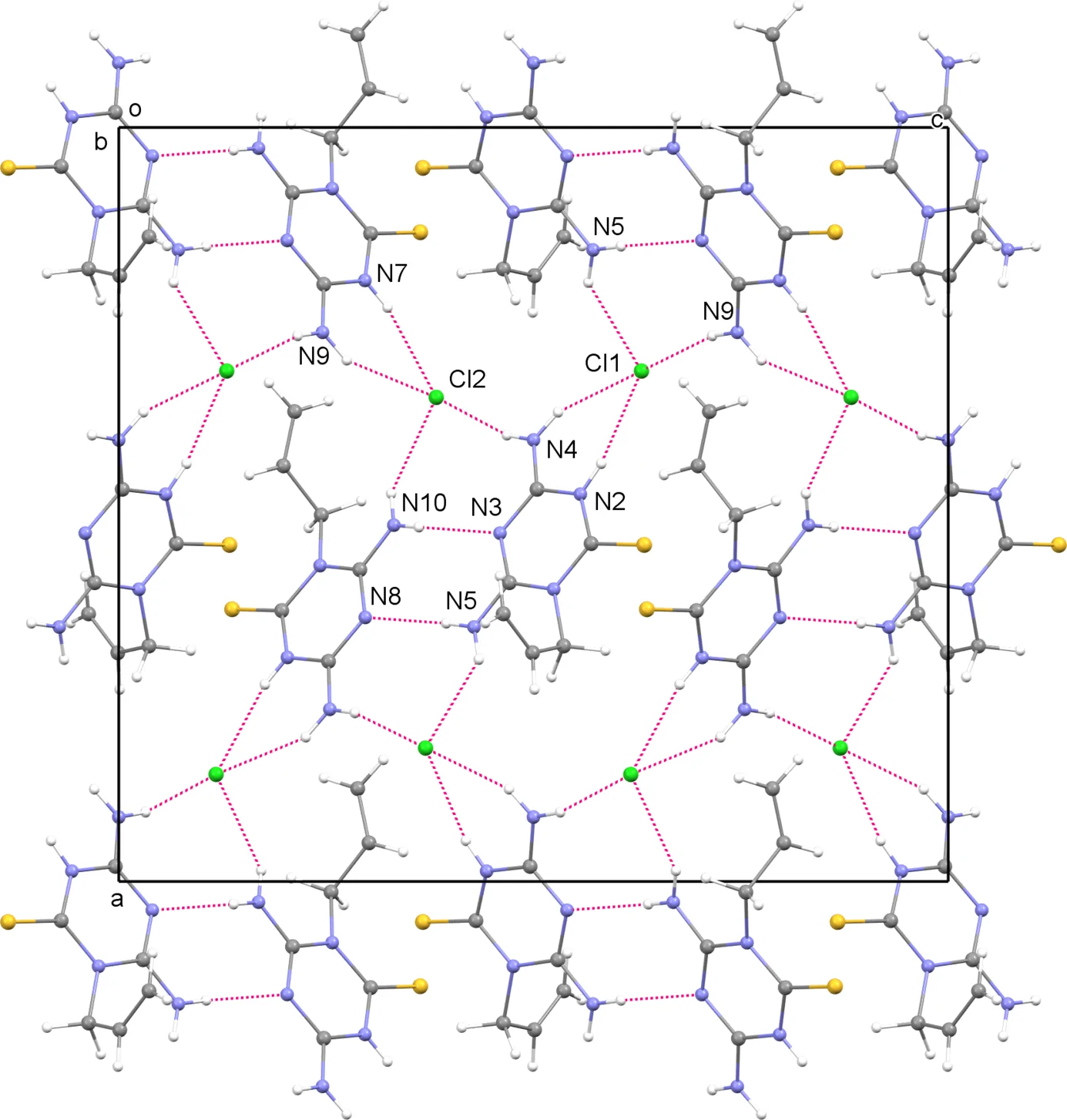
A segment of the crystal structure viewed along the *b*-axis direction with N—H⋯N and N—H⋯Cl hydrogen-bond inter­actions shown as dotted lines. For clarity, only one layer of the structure is depicted.

In the crystal, the two independent cations are linked by a pair of N—H⋯N hydrogen bonds, namely N10—H10*C*⋯N3 and N5—H5*A*⋯N8, in which the amino groups are the donors and the triazine nitro­gen atoms are the acceptors, forming 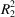
(8) motifs (Table 1 ▸, Fig. 2 ▸). The resultant hydrogen-bonded cation pairs are bridged by chloride anions through N—H⋯Cl contacts to neighbouring pairs. Each of the two independent chloride anions accepts four N—H⋯Cl contacts of which three are from amino groups and one from a triazine N—H group.

**Figure 3:**
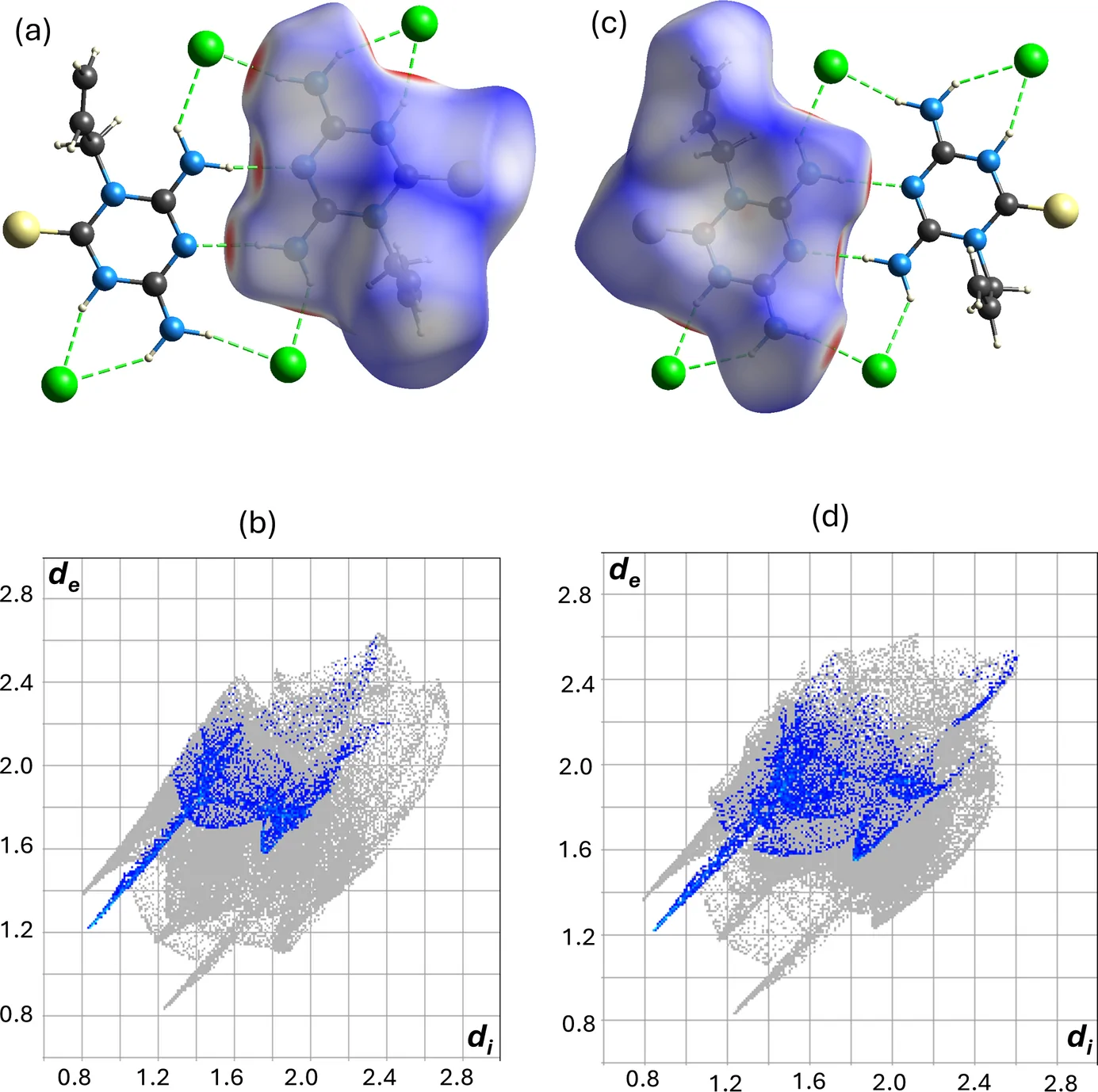
(*a*) The *d*_norm_ Hirshfeld surface of cation **c1** showing N—H⋯N and N—H⋯Cl hydrogen bond contacts as green dashed lines. (*b*) The Hirshfeld surface two-dimensional fingerprint plot for **c1** with inter­molecular contacts involving nitro­gen atoms highlighted in blue. (*c*) The *d*_norm_ Hirshfeld surface of cation **c2** showing N—H⋯N and N—H⋯Cl hydrogen-bond contacts as green dashed lines. (*d*) The Hirshfeld surface two-dimensional fingerprint plots for **c2** with inter­molecular contacts involving nitro­gen atoms highlighted in blue.

The Hirshfeld surfaces obtained using *CrystalExplorer* (Spackman *et al.*, 2021 ▸) illustrate the similarities in the closest inter­molecular contacts for the two independent cations (Fig. 3 ▸*a* for **c1** and Fig. 3 ▸*c* for **c2**). Surface coverage analysis by element shows H (68.3%), N (11.4%), S (12.9%) and C (7.4%) for **c1** and H (66.6%), N (12.1%), S (12.7%) and C (8.5%) for **c2**. The results show that the independent cations are involved in similar inter­molecular contacts with their neighbours. However, the inter­molecular contacts are not identical, as shown by examination of the two-dimensional fingerprint plots for cations **c1** (Fig. 3 ▸*b*) and **c2** (Fig. 3 ▸*d*).

## 4. Database survey

A search of the Cambridge Structural Database (CSD, updated to April 2026; Groom, *et al.*, 2016 ▸) for the DTT group gave two hits. In 4,6-di­amino-1-cyclo­hexyl-1,3,5-triazine-2(1*H*)-thione monohydrate (CSD refcode AZAZUA; Ahmed *et al.*, 2026 ▸), the DTT group is bonded to a cyclo­hexane ring and hydrogen bonded by water. Bis(4,6-di­amino-2-thiono-1*H*-(1,3,5)triazinium) aqua­bis­(oxalato-*O*,*O*′)dioxo­uranium(VI) *N*-cyano­guanidine (QELQAA) (Serezhkina *et al.*, 2007 ▸) has two independent DTT groups and displays an 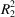
(8) motif akin to that observed in the title compound.

## 5. Synthesis and crystallization

**Figure 4:**
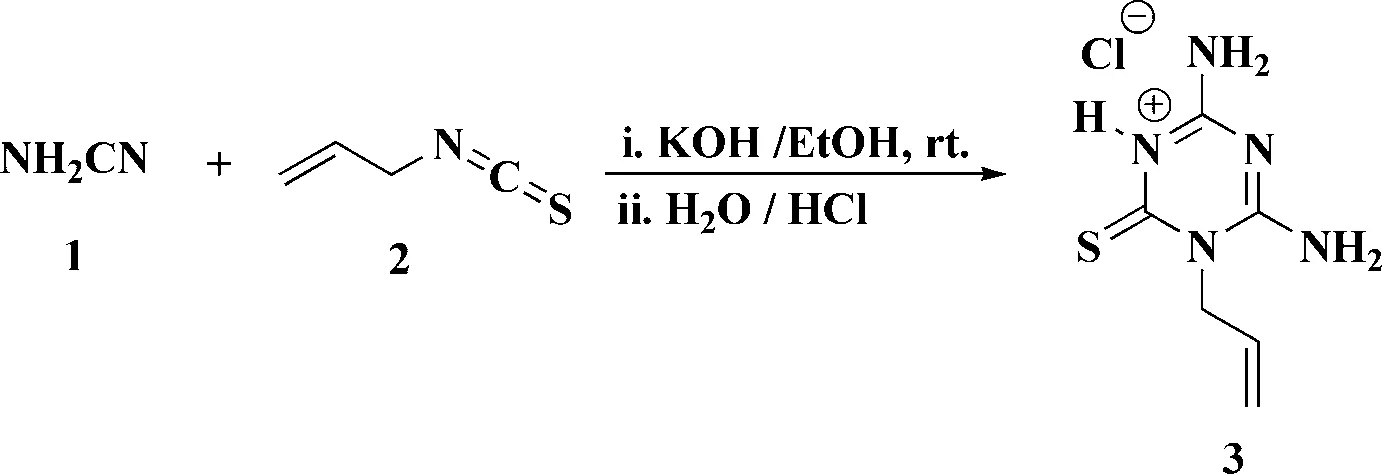
Reaction scheme for the formation of the title compound.

The title compound was obtained, as shown in Fig. 4 ▸, from the reaction of cyanamide (**1**) with allyl­iso­thio-cyanate (**2**) in ethanol in the presence of potassium hydroxide at room temperature for 30 min. The reaction mixture was then poured on water and hydrolysed using hydro­chloric acid, , then recrystallized from aqueous solution to afford the title compound **3**.

## 6. Refinement

Crystal data, data collection and structure refinement details are summarized in Table 2 ▸. The N-bound H atoms were freely refined. Other H atoms were place in idealized position and refined using a riding model with *U*_iso_(H) = 1.2 or 1.5*U*_eq_(C).

## Supplementary Material

Crystal structure: contains datablock(s) I. DOI: 10.1107/S2056989026008182/jp2031sup1.cif

Structure factors: contains datablock(s) I. DOI: 10.1107/S2056989026008182/jp2031Isup2.hkl

Supporting information file. DOI: 10.1107/S2056989026008182/jp2031Isup3.cml

CCDC reference: 2570884

Additional supporting information:  crystallographic information; 3D view; checkCIF report

## Figures and Tables

**Table 1:** Hydrogen-bond geometry (Å, °)

*D*—H⋯*A*	*D*—H	H⋯*A*	*D*⋯*A*	*D*—H⋯*A*
C10—H10*B*⋯S1^i^	0.97	3.01	3.675 (3)	127
N4—H4*C*⋯Cl2^ii^	0.85 (1)	2.34 (1)	3.183 (2)	169 (3)
N4—H4*D*⋯Cl1	0.86 (1)	2.44 (2)	3.243 (2)	155 (2)
N5—H5*A*⋯N8	0.86 (1)	2.21 (1)	3.070 (3)	172 (3)
N5—H5*B*⋯Cl1^iii^	0.86 (1)	2.36 (2)	3.148 (2)	153 (3)
N9—H9*A*⋯Cl1^iii^	0.86 (1)	2.47 (1)	3.314 (2)	171 (3)
N9—H9*B*⋯Cl2	0.86 (1)	2.64 (2)	3.400 (2)	148 (3)
N10—H10*C*⋯N3	0.86 (1)	2.22 (1)	3.056 (3)	163 (3)
N10—H10*D*⋯Cl2^ii^	0.86 (1)	2.42 (2)	3.142 (2)	143 (3)
N7—H7⋯Cl2	0.83 (3)	2.34 (3)	3.131 (2)	161 (3)
N2—H2⋯Cl1	0.86 (4)	2.33 (4)	3.144 (2)	158 (3)

Symmetry codes: (i) 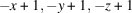; (ii) 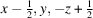; (iii) 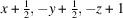.

**Table 2:** Experimental details

Crystal data	
Chemical formula	C_6_H_10_N_5_S^+^·Cl^−^
*M*_r_	219.70
Crystal system, space group	Orthorhombic, *Pbca*
Temperature (K)	296
*a*, *b*, *c* (Å)	17.2243 (4), 12.2458 (4), 18.9429 (5)
*V* (Å^3^)	3995.54 (19)
*Z*	16
Radiation type	Cu *K*α
μ (mm^−1^)	5.05
Crystal size (mm)	0.14 × 0.09 × 0.07

Data collection	
Diffractometer	SuperNova, Dual, Cu at home/near, Atlas
Absorption correction	Gaussian (*CrysAlis PRO*; Rigaku OD, 2026 ▸)
*T*_min_, *T*_max_	0.605, 0.799
No. of measured, independent and observed [*I* > 2σ(*I*)] reflections	14045, 3733, 3093
*R*_int_	0.037
(sin θ/λ)_max_ (Å^−1^)	0.610

Refinement	
*R*[*F*^2^ > 2σ(*F*^2^)], *wR*(*F*^2^), *S*	0.041, 0.125, 1.06
No. of reflections	3733
No. of parameters	275
No. of restraints	8
H-atom treatment	H atoms treated by a mixture of independent and constrained refinement
Δρ_max_, Δρ_min_ (e Å^−3^)	0.30, −0.61

Computer programs: *CrysAlis PRO* (Rigaku OD, 2026 ▸), *SHELXT* (Sheldrick, 2015*a* ▸), *SHELXL* (Sheldrick, 2015*b* ▸), *ORTEP-3 for Windows* (Farrugia, 2012 ▸) and *Mercury* (Macrae *et al.*, 2020 ▸)..

## supplementary crystallographic information

### 4,6-Diamino-3-(prop-2-en-1-yl)-2-sulfanylidene-2,3-dihydro-1,3,5-triazin-1-ium chloride Crystal data

**Table:**

C_6_H_10_N_5_S^+^·Cl^−^	*D*_x_ = 1.461 Mg m^−^^3^
*M**_r_* = 219.70	Cu *K*α radiation, λ = 1.54184 Å
Orthorhombic, *P**b**c**a*	Cell parameters from 6530 reflections
*a* = 17.2243 (4) Å	θ = 4.7–70.0°
*b* = 12.2458 (4) Å	µ = 5.05 mm^−^^1^
*c* = 18.9429 (5) Å	*T* = 296 K
*V* = 3995.54 (19) Å^3^	Block, colourless
*Z* = 16	0.14 × 0.09 × 0.07 mm
*F*(000) = 1824	

### 4,6-Diamino-3-(prop-2-en-1-yl)-2-sulfanylidene-2,3-dihydro-1,3,5-triazin-1-ium chloride Data collection

**Table:**

SuperNova, Dual, Cu at home/near, Atlas diffractometer	3093 reflections with *I* > 2σ(*I*)
Detector resolution: 10.5082 pixels mm^-1^	*R*_int_ = 0.037
ω scans	θ_max_ = 70.2°, θ_min_ = 4.7°
Absorption correction: gaussian (CrysAlisPro; Rigaku OD, 2026)	*h* = −20→20
*T*_min_ = 0.605, *T*_max_ = 0.799	*k* = −10→14
14045 measured reflections	*l* = −15→22
3733 independent reflections	

### 4,6-Diamino-3-(prop-2-en-1-yl)-2-sulfanylidene-2,3-dihydro-1,3,5-triazin-1-ium chloride Refinement

**Table:**

Refinement on *F*^2^	8 restraints
Least-squares matrix: full	Hydrogen site location: mixed
*R*[*F*^2^ > 2σ(*F*^2^)] = 0.041	H atoms treated by a mixture of independent and constrained refinement
*wR*(*F*^2^) = 0.125	*w* = 1/[σ^2^(*F*_o_^2^) + (0.0689*P*)^2^ + 1.5902*P*] where *P* = (*F*_o_^2^ + 2*F*_c_^2^)/3
*S* = 1.06	(Δ/σ)_max_ = 0.001
3733 reflections	Δρ_max_ = 0.30 e Å^−^^3^
275 parameters	Δρ_min_ = −0.61 e Å^−^^3^

### 4,6-Diamino-3-(prop-2-en-1-yl)-2-sulfanylidene-2,3-dihydro-1,3,5-triazin-1-ium chloride Special details

**Table:**

Geometry. All esds (except the esd in the dihedral angle between two l.s. planes) are estimated using the full covariance matrix. The cell esds are taken into account individually in the estimation of esds in distances, angles and torsion angles; correlations between esds in cell parameters are only used when they are defined by crystal symmetry. An approximate (isotropic) treatment of cell esds is used for estimating esds involving l.s. planes.

### 4,6-Diamino-3-(prop-2-en-1-yl)-2-sulfanylidene-2,3-dihydro-1,3,5-triazin-1-ium chloride Fractional atomic coordinates and isotropic or equivalent isotropic displacement parameters (Å^2^)

**Table:**

	*x*	*y*	*z*	*U*_iso_*/*U*_eq_	
C1	0.55186 (14)	0.1964 (2)	0.56942 (12)	0.0416 (5)	
C2	0.47990 (13)	0.33028 (18)	0.50421 (11)	0.0339 (5)	
C3	0.60373 (13)	0.29380 (19)	0.47078 (11)	0.0371 (5)	
C4	0.68763 (15)	0.1641 (2)	0.53638 (15)	0.0512 (6)	
H4A	0.728925	0.212943	0.521743	0.061*	
H4B	0.693712	0.151018	0.586580	0.061*	
C5	0.6966 (2)	0.0598 (3)	0.4988 (2)	0.0726 (9)	
H5	0.745325	0.027360	0.501020	0.087*	
C6	0.6451 (3)	0.0090 (4)	0.4635 (3)	0.0966 (13)	
H6A	0.595291	0.037773	0.459533	0.116*	
H6B	0.657227	−0.056859	0.441733	0.116*	
C7	0.64075 (13)	0.6258 (2)	0.19641 (12)	0.0396 (5)	
C8	0.70801 (13)	0.47868 (19)	0.25284 (11)	0.0367 (5)	
C9	0.58648 (13)	0.5205 (2)	0.29058 (11)	0.0367 (5)	
C10	0.51400 (15)	0.6800 (2)	0.24412 (14)	0.0515 (6)	
H10A	0.531011	0.751773	0.228941	0.062*	
H10B	0.495489	0.686428	0.292308	0.062*	
C11	0.44879 (18)	0.6440 (3)	0.19837 (17)	0.0664 (8)	
H11	0.460874	0.606170	0.157231	0.080*	
C12	0.3768 (2)	0.6616 (4)	0.2120 (2)	0.0829 (11)	
H12A	0.362798	0.699195	0.252670	0.100*	
H12B	0.338726	0.636846	0.181065	0.100*	
Cl1	0.32306 (3)	0.26965 (5)	0.63015 (3)	0.04541 (18)	
Cl2	0.85800 (3)	0.55220 (5)	0.11729 (3)	0.04427 (18)	
N1	0.61244 (11)	0.21857 (16)	0.52437 (10)	0.0375 (4)	
N2	0.48631 (12)	0.25456 (17)	0.55655 (11)	0.0408 (5)	
N3	0.53805 (11)	0.34843 (16)	0.45961 (9)	0.0364 (4)	
N4	0.41451 (11)	0.38403 (18)	0.50004 (11)	0.0420 (5)	
N5	0.66255 (12)	0.3135 (2)	0.42838 (12)	0.0469 (5)	
N6	0.58105 (11)	0.60458 (17)	0.24269 (10)	0.0374 (4)	
N7	0.70284 (12)	0.55877 (17)	0.20332 (11)	0.0403 (4)	
N8	0.64983 (11)	0.45932 (16)	0.29709 (10)	0.0374 (4)	
N9	0.77166 (13)	0.4203 (2)	0.25436 (13)	0.0532 (6)	
N10	0.52738 (13)	0.4994 (2)	0.33153 (12)	0.0504 (6)	
S1	0.55368 (5)	0.10760 (7)	0.63339 (4)	0.0687 (3)	
S2	0.63898 (5)	0.72293 (7)	0.13656 (4)	0.0632 (2)	
H4C	0.4060 (17)	0.4314 (18)	0.4679 (11)	0.051 (8)*	
H4D	0.3786 (12)	0.366 (2)	0.5294 (11)	0.042 (7)*	
H5A	0.6553 (19)	0.357 (2)	0.3935 (11)	0.054 (9)*	
H5B	0.7048 (11)	0.276 (2)	0.4250 (17)	0.060 (9)*	
H9A	0.7791 (18)	0.3688 (18)	0.2842 (13)	0.055 (8)*	
H9B	0.8075 (15)	0.433 (3)	0.2238 (14)	0.068 (10)*	
H10C	0.5313 (19)	0.4462 (17)	0.3609 (13)	0.053 (9)*	
H10D	0.4852 (12)	0.537 (2)	0.3300 (17)	0.065 (10)*	
H7	0.7391 (19)	0.570 (2)	0.1757 (16)	0.053 (8)*	
H2	0.446 (2)	0.242 (3)	0.5818 (19)	0.073 (11)*	

### 4,6-Diamino-3-(prop-2-en-1-yl)-2-sulfanylidene-2,3-dihydro-1,3,5-triazin-1-ium chloride Atomic displacement parameters (Å^2^)

**Table:**

	*U*^11^	*U*^22^	*U*^33^	*U*^12^	*U*^13^	*U*^23^
C1	0.0452 (13)	0.0414 (13)	0.0383 (11)	0.0041 (10)	−0.0003 (10)	0.0004 (10)
C2	0.0358 (11)	0.0349 (12)	0.0309 (10)	−0.0011 (9)	−0.0007 (8)	−0.0004 (9)
C3	0.0356 (11)	0.0414 (12)	0.0343 (11)	0.0005 (9)	−0.0027 (9)	−0.0055 (9)
C4	0.0384 (13)	0.0595 (17)	0.0559 (15)	0.0070 (12)	−0.0062 (11)	0.0073 (13)
C5	0.0529 (17)	0.0582 (19)	0.107 (3)	0.0196 (15)	0.0048 (18)	0.0043 (19)
C6	0.101 (3)	0.070 (2)	0.118 (3)	0.017 (2)	0.001 (3)	−0.031 (2)
C7	0.0375 (12)	0.0419 (13)	0.0395 (11)	−0.0004 (10)	−0.0023 (9)	0.0003 (10)
C8	0.0332 (11)	0.0403 (12)	0.0367 (11)	0.0019 (9)	0.0012 (9)	0.0020 (9)
C9	0.0319 (11)	0.0445 (13)	0.0336 (11)	−0.0006 (9)	−0.0013 (8)	−0.0022 (9)
C10	0.0466 (14)	0.0563 (16)	0.0515 (14)	0.0168 (12)	0.0022 (11)	0.0026 (12)
C11	0.0511 (16)	0.090 (2)	0.0584 (17)	0.0188 (16)	−0.0088 (13)	0.0073 (16)
C12	0.0570 (18)	0.107 (3)	0.085 (2)	0.0038 (19)	−0.0070 (17)	0.036 (2)
Cl1	0.0348 (3)	0.0570 (4)	0.0445 (3)	−0.0086 (2)	0.0021 (2)	0.0004 (3)
Cl2	0.0355 (3)	0.0570 (4)	0.0402 (3)	0.0050 (2)	0.0046 (2)	−0.0017 (2)
N1	0.0359 (9)	0.0390 (10)	0.0375 (9)	0.0042 (8)	−0.0031 (8)	0.0002 (8)
N2	0.0376 (10)	0.0451 (11)	0.0396 (10)	0.0020 (9)	0.0051 (9)	0.0084 (9)
N3	0.0326 (9)	0.0436 (11)	0.0330 (9)	0.0015 (8)	0.0014 (7)	0.0026 (8)
N4	0.0357 (10)	0.0507 (13)	0.0395 (10)	0.0052 (9)	0.0042 (8)	0.0088 (9)
N5	0.0350 (10)	0.0612 (14)	0.0444 (11)	0.0069 (10)	0.0058 (9)	0.0069 (10)
N6	0.0328 (9)	0.0448 (11)	0.0347 (9)	0.0047 (8)	−0.0012 (7)	0.0002 (8)
N7	0.0331 (10)	0.0477 (12)	0.0401 (10)	0.0012 (9)	0.0071 (8)	0.0072 (9)
N8	0.0332 (9)	0.0413 (11)	0.0375 (9)	0.0028 (8)	0.0033 (8)	0.0044 (8)
N9	0.0400 (11)	0.0607 (15)	0.0589 (13)	0.0139 (10)	0.0140 (10)	0.0191 (11)
N10	0.0363 (11)	0.0702 (16)	0.0448 (12)	0.0084 (11)	0.0094 (9)	0.0136 (11)
S1	0.0762 (5)	0.0689 (5)	0.0611 (4)	0.0200 (4)	0.0068 (4)	0.0251 (4)
S2	0.0600 (5)	0.0636 (5)	0.0660 (5)	0.0049 (3)	0.0037 (3)	0.0287 (4)

### 4,6-Diamino-3-(prop-2-en-1-yl)-2-sulfanylidene-2,3-dihydro-1,3,5-triazin-1-ium chloride Geometric parameters (Å, º)

**Table:**

C1—N2	1.357 (3)	C8—N7	1.360 (3)
C1—N1	1.375 (3)	C9—N10	1.306 (3)
C1—S1	1.629 (2)	C9—N8	1.329 (3)
C2—N4	1.307 (3)	C9—N6	1.375 (3)
C2—N3	1.329 (3)	C10—N6	1.479 (3)
C2—N2	1.362 (3)	C10—C11	1.486 (4)
C3—N5	1.315 (3)	C10—H10A	0.9700
C3—N3	1.331 (3)	C10—H10B	0.9700
C3—N1	1.379 (3)	C11—C12	1.284 (5)
C4—C5	1.470 (5)	C11—H11	0.9300
C4—N1	1.475 (3)	C12—H12A	0.9300
C4—H4A	0.9700	C12—H12B	0.9300
C4—H4B	0.9700	N2—H2	0.86 (4)
C5—C6	1.273 (6)	N4—H4C	0.854 (10)
C5—H5	0.9300	N4—H4D	0.860 (10)
C6—H6A	0.9300	N5—H5A	0.861 (10)
C6—H6B	0.9300	N5—H5B	0.861 (10)
C7—N7	1.355 (3)	N7—H7	0.83 (3)
C7—N6	1.376 (3)	N9—H9A	0.857 (10)
C7—S2	1.643 (2)	N9—H9B	0.860 (10)
C8—N9	1.309 (3)	N10—H10C	0.859 (10)
C8—N8	1.328 (3)	N10—H10D	0.860 (10)
			
N2—C1—N1	114.6 (2)	C11—C10—H10B	108.9
N2—C1—S1	120.01 (19)	H10A—C10—H10B	107.8
N1—C1—S1	125.36 (19)	C12—C11—C10	124.2 (4)
N4—C2—N3	121.8 (2)	C12—C11—H11	117.9
N4—C2—N2	117.2 (2)	C10—C11—H11	117.9
N3—C2—N2	121.0 (2)	C11—C12—H12A	120.0
N5—C3—N3	117.7 (2)	C11—C12—H12B	120.0
N5—C3—N1	119.2 (2)	H12A—C12—H12B	120.0
N3—C3—N1	123.0 (2)	C1—N1—C3	120.4 (2)
C5—C4—N1	114.2 (2)	C1—N1—C4	118.8 (2)
C5—C4—H4A	108.7	C3—N1—C4	120.8 (2)
N1—C4—H4A	108.7	C1—N2—C2	123.7 (2)
C5—C4—H4B	108.7	C1—N2—H2	119 (2)
N1—C4—H4B	108.7	C2—N2—H2	118 (2)
H4A—C4—H4B	107.6	C2—N3—C3	117.08 (19)
C6—C5—C4	127.3 (3)	C2—N4—H4C	122 (2)
C6—C5—H5	116.4	C2—N4—H4D	117.1 (19)
C4—C5—H5	116.4	H4C—N4—H4D	121 (3)
C5—C6—H6A	120.0	C3—N5—H5A	118 (2)
C5—C6—H6B	120.0	C3—N5—H5B	127 (2)
H6A—C6—H6B	120.0	H5A—N5—H5B	113 (3)
N7—C7—N6	114.5 (2)	C9—N6—C7	120.71 (19)
N7—C7—S2	121.33 (18)	C9—N6—C10	120.56 (19)
N6—C7—S2	124.21 (18)	C7—N6—C10	118.5 (2)
N9—C8—N8	121.4 (2)	C7—N7—C8	123.7 (2)
N9—C8—N7	117.6 (2)	C7—N7—H7	116 (2)
N8—C8—N7	121.0 (2)	C8—N7—H7	120 (2)
N10—C9—N8	118.3 (2)	C8—N8—C9	117.41 (19)
N10—C9—N6	119.1 (2)	C8—N9—H9A	123 (2)
N8—C9—N6	122.6 (2)	C8—N9—H9B	119 (2)
N6—C10—C11	113.2 (2)	H9A—N9—H9B	118 (3)
N6—C10—H10A	108.9	C9—N10—H10C	118 (2)
C11—C10—H10A	108.9	C9—N10—H10D	122 (2)
N6—C10—H10B	108.9	H10C—N10—H10D	120 (3)
			
N1—C4—C5—C6	−6.9 (6)	N1—C3—N3—C2	2.0 (3)
N6—C10—C11—C12	147.5 (3)	N10—C9—N6—C7	−176.9 (2)
N2—C1—N1—C3	−0.7 (3)	N8—C9—N6—C7	3.1 (3)
S1—C1—N1—C3	178.38 (18)	N10—C9—N6—C10	8.6 (3)
N2—C1—N1—C4	176.7 (2)	N8—C9—N6—C10	−171.4 (2)
S1—C1—N1—C4	−4.3 (3)	N7—C7—N6—C9	−0.6 (3)
N5—C3—N1—C1	−180.0 (2)	S2—C7—N6—C9	178.94 (18)
N3—C3—N1—C1	0.1 (3)	N7—C7—N6—C10	174.0 (2)
N5—C3—N1—C4	2.7 (3)	S2—C7—N6—C10	−6.4 (3)
N3—C3—N1—C4	−177.2 (2)	C11—C10—N6—C9	−91.0 (3)
C5—C4—N1—C1	90.8 (3)	C11—C10—N6—C7	94.4 (3)
C5—C4—N1—C3	−91.8 (3)	N6—C7—N7—C8	−1.9 (3)
N1—C1—N2—C2	−0.9 (3)	S2—C7—N7—C8	178.58 (19)
S1—C1—N2—C2	180.00 (19)	N9—C8—N7—C7	−179.2 (2)
N4—C2—N2—C1	−176.7 (2)	N8—C8—N7—C7	2.0 (4)
N3—C2—N2—C1	3.1 (4)	N9—C8—N8—C9	−178.2 (2)
N4—C2—N3—C3	176.3 (2)	N7—C8—N8—C9	0.5 (3)
N2—C2—N3—C3	−3.5 (3)	N10—C9—N8—C8	177.0 (2)
N5—C3—N3—C2	−177.9 (2)	N6—C9—N8—C8	−3.0 (3)

### 4,6-Diamino-3-(prop-2-en-1-yl)-2-sulfanylidene-2,3-dihydro-1,3,5-triazin-1-ium chloride Hydrogen-bond geometry (Å, º)

**Table:**

*D*—H···*A*	*D*—H	H···*A*	*D*···*A*	*D*—H···*A*
C10—H10*B*···S1^i^	0.97	3.01	3.675 (3)	127
N4—H4*C*···Cl2^ii^	0.85 (1)	2.34 (1)	3.183 (2)	169 (3)
N4—H4*D*···Cl1	0.86 (1)	2.44 (2)	3.243 (2)	155 (2)
N5—H5*A*···N8	0.86 (1)	2.21 (1)	3.070 (3)	172 (3)
N5—H5*B*···Cl1^iii^	0.86 (1)	2.36 (2)	3.148 (2)	153 (3)
N9—H9*A*···Cl1^iii^	0.86 (1)	2.47 (1)	3.314 (2)	171 (3)
N9—H9*B*···Cl2	0.86 (1)	2.64 (2)	3.400 (2)	148 (3)
N10—H10*C*···N3	0.86 (1)	2.22 (1)	3.056 (3)	163 (3)
N10—H10*D*···Cl2^ii^	0.86 (1)	2.42 (2)	3.142 (2)	143 (3)
N7—H7···Cl2	0.83 (3)	2.34 (3)	3.131 (2)	161 (3)
N2—H2···Cl1	0.86 (4)	2.33 (4)	3.144 (2)	158 (3)

Symmetry codes: (i) −*x*+1, −*y*+1, −*z*+1; (ii) *x*−1/2, *y*, −*z*+1/2; (iii) *x*+1/2, −*y*+1/2, −*z*+1.
